# The Serendipity Case of the Pedunculopontine Nucleus Low-Frequency Brain Stimulation: Chasing a Gait Response, Finding Sleep, and Cognition Improvement

**DOI:** 10.3389/fneur.2013.00068

**Published:** 2013-06-05

**Authors:** Alessandro Stefani, Antonella Peppe, Salvatore Galati, Mario Stampanoni Bassi, Vincenza D’Angelo, Mariangela Pierantozzi

**Affiliations:** ^1^Department of Neuroscience, “Tor Vergata” University, Rome, Italy; ^2^IRCCS, Fondazione Santa Lucia, Rome, Italy; ^3^Neurocenter of Southern Switzerland, Lugano, Switzerland

**Keywords:** deep brain stimulation, Parkinson disease, neuromodulation, executive function, sleep structure

## Abstract

Deep brain stimulation (DBS) of the subthalamic nucleus (STN) is an efficacious therapy for Parkinson’s disease (PD) but its effects on non-motor facets may be detrimental. The low-frequency stimulation (LFS) of the pedunculopontine nucleus (PPN or the nucleus tegmenti pedunculopontini – PPTg-) opened new perspectives. In our hands, PPTg-LFS revealed a modest influence on gait but increased sleep quality and degree of attentiveness. At odds with potential adverse events following STN-DBS, executive functions, under PPTg-ON, ameliorated. A recent study comparing both targets found that only PPTg-LFS improved night-time sleep and daytime sleepiness. Chances are that different neurosurgical groups influence either the PPN sub-portion identified as pars dissipata (more interconnected with GPi/STN) or the caudal PPN region known as pars compacta, preferentially targeting intralaminar and associative nucleus of the thalamus. Yet, the wide electrical field delivered affects a plethora of en passant circuits, and a fine distinction on the specific pathways involved is elusive. This review explores our angle of vision, by which PPTg-LFS activates cholinergic and glutamatergic ascending fibers, influencing non-motor behaviors.

## State-of-the-Art of Motor and Non-Motor Effects of PPN-DBS

Deep brain stimulation (DBS) of the Subthalamic nucleus (STN) has acquired the status of reliable therapy for Parkinson’s disease (PD) patients (Benabid et al., 2009; Moro et al., 2010a). However, in recent years, the neurosurgical treatment of movement disorders has been focused on identifying alternative targets to the traditional STN, in order to minimize adverse events and to rescue motor axial signs (Pahapill and Lozano, 2000; Nandi et al., 2002b; Mena-Segovia et al., 2004; Stefani et al., 2007). In fact, although the efficacy of STN-DBS on segmental motor symptoms is proved, other motor features (akinesia, freezing of gait) are relatively resistant to STN-DBS or may even worsen (Kleiner-Fisman et al., 2003; Rodriguez-Oroz et al., 2005). Incidentally, the benefits promoted by DBS of the ventromedial tiers of the globus pallidus internus (GPi), too rapidly abandoned in the nineties, were similar to those obtained by STN-DBS, and were scarcely impressive on gait (Rodriguez-Oroz et al., 2005; Follett et al., 2010).

The pedunculopontine nucleus area (PPN-a) represents an heterogeneous structure placed in the dorso-lateral mesopontine tegmentum; it consists of a pars *compacta* and a pars *dissipata* and features a complex cells organization, which engages different population of cholinergic, glutamatergic, and GABAergic neurons (Mena-Segovia et al., 2009; Wang and Morales, 2009). As known, PPN-a, part of the reticular activating system, plays a critical role in regulating the sleep-awake cycle and facilitating arousal mechanisms (Steriade et al., 1990a; Rye, 1997; Datta, 2002). PPN-a is also strongly involved in other facets of behavior including motivation, attention, reward, and mnemonic processes (Steckler et al., 1994; Winn, 2006; Andero et al., 2007; Ros et al., 2010).

In addition, it has been largely inferred that portions of PPN-a participates in locomotion and contributes to postural tone as a result of the influence on the lower brainstem and on the spinal cord, exerted via the pontomedullary reticular formation and reticulospinal nuclei (Lee et al., 2000; Pahapill and Lozano, 2000; Takakusaki et al., 2003; Mena-Segovia et al., 2004; Pierantozzi et al., 2008). Experimental models proved that “some dorsal tiers” of the PPN-a, i.e., the traditional mesencephalic locomotor region, induces initiation, and maintenance of locomotion (Garcia-Rill et al., 1987; Skinner et al., 1990; Takakusaki, 2008), while its inactivation, by lesion or high frequency stimulation (HFS), produces akinesia in non-human primates (Munro-Davies et al., 1999; Nandi et al., 2002b). It should be noted that this form of experimental akinesia may be reverted by the low-frequency stimulation (LFS) of PPN-a (Nandi et al., 2002a,b; Jenkinson et al., 2004). Moreover, extensive degeneration of PPN-a occurs in idiopathic PD (Hirsch et al., 1987; Pahapill and Lozano, 2000). An adjourned excursus of the complex functional organization of PPN region is available in the review by Benarroch (2013).

All this considered, PPN-a was envisioned as a good and safe candidate for stereotactic neurosurgery in PD patients featuring prominent and levodopa-resistant axial impairment (Pahapill and Lozano, 2000; Mena-Segovia et al., 2004; Pereira et al., 2008). The pioneering works were performed by an Italian and a British group (Mazzone et al., 2005; Plaha and Gill, 2005). Mazzone has renamed further his preferential target as the nucleus tegmenti pedunculopontini (PPTg) (Mazzone et al., 2007, 2008, 2011). For reasons of clarity, from now on, the definition PPTg will be utilized for works produced by the Italian specialists, whilst PPN-a will be maintained for all the others.

Since then, other groups successfully completed the neurosurgical procedure targeting either bilateral or mono-lateral PPN-a, in limited series of selected PD patients. Table 1 illustrates most of the available works worldwide (choice was not limited to, but oriented toward, those manuscripts capable to illustrate also non-motor domains).

**Table 1:** **Selected papers on PD patients implanted in the “PPN-area” (estimated 60 patients from Rome, Bristol, Oxford, Melbourne, Grenoble, Toronto)**.

Selected papers	Patients	DBS targeting (and follow-up)	Motor symptoms	Sleep and cognitive domains
Mazzone et al. (2005)	*N* = 2	Bilateral PPTg and STN (1 month)	SAFETY study	NA
Plaha and Gill (2005)	*N* = 2	Bilateral PPN (3 months)	Improvement of gait dysfunction and postural instability	NA
Stefani et al. (2007)	*N* = 6	Bilateral PPTg and STN (6 months)	Significant improvement	NA (but surprising intra-operative *pleasant arousal*)
Lim et al. (2007)	*N* = 1	Unilat. PPN (acute)	N-AP	Intrapontine and scalp EEG recordings: evidence of ponto-geniculo-occipital (PGO) waves in humans
Romigi et al. (2008)	*N* = 1	Bilateral PPTg and STN	N-AP	First polysomnographic (PSG) recordings: relevant increase in REM sleep (note: from the same series of Stefani et al., 2007)
Lim et al. (2009)	3 PD;2 PSP	Unilat. PPN (post-surgery)	N-AP	PSG study: strong modulation of REM sleep
Zanini et al. (2009)	*N* = 5	Bilateral PPTg and STN (1 year)	(See Stefani et al., 2007)	Improvement of the grammatical aspect of language (Note: from the same series of Stefani et al., 2007)
Ceravolo et al. (2011)	*N* = 6	Bilateral PPTg and STN (1 year)	(See Stefani et al., 2007)	Improved executive functions; increased FDG consumption in prefrontal areas and mono-lateral ventral striatum (see Figure 2)
Peppe et al. (2010)	*N* = 5	Bilateral PPTg and STN (1 year)	Gait analysis: effects on kinematics and spatio-temporal variables	NA (note: from the same series of Stefani et al., 2007)
Moro et al. (2010b)	*N* = 6	Unilat. PPN (3–12 months)	Significant reduction in falls	NA
Ferraye et al. (2010)	*N* = 6	Bilateral PPN (plus *previous* STN) (4–12 months)	Variable effects on gait disorder	NA
Arnulf et al. (2010)	*N* = 2 (1-year follow up)	Bilateral PPN (on *previous* STN)	Improvement of FOG and falls	LFS increases alertness, HFS induces non-REM sleep. The sudden withdrawal of LFS is followed by REM sleep episodes (note: from the same series of Ferraye)
Strafella et al. (2008)	*N* = 1	Unilat. PPN (3 months)	Improved motor function	PET study: significant increase of rCBF in sub-cortical areas, mostly thalamus, bilaterally
Ostrem et al. (2010)	*N* = 1 (PPFG)	Bilateral PPN (1 year)	Only mild improvement of freezing and gait impairment	NA
Costa et al. (2010)	*N* = 5	Bilateral PPTg and STN (3 months)	29% reduction of UPDRS part-III score	Significant improvement in working memory (note: same cohort as Stefani et al., 2007)
Wilcox et al. (2011)	*N* = 1 (*PPFG*)	Bilateral PPN	Robust improvement of gait and posture	NA
Pierantozzi et al. (2008)	*N* = 6	Bilateral PPTg and STN (3–6 months)	Hoffman Reflex-Th. increase	NA (note: same cohort as Stefani et al., 2007)
Mazzone et al. (2011)	*N* = 17	13 Unilat. PPTg, 4 PPTg plus GPi		NA
Brusa et al. (2009)	1 PSP	Unilat. PPTg (9 months)	Negligible	Marginal effect on cognitive domains (only minimal improvement in verbal fluency)
Androulidakis et al. (2008)	*N* = 6	2 Bilateral PPN and STN	NA; intrasurgical neurophysiology	7-11 Hz oscillatory synchronization in PPN coupled with cortical alpha
		2 Unilat. PPN and STN	
		1 Unilat. GPi and PPN	
		1 Unilat. PPN alone	
Shimamoto et al. (2010)	2 PD, 2 PSP			?
Thevathasan et al. (2011)	5 PD with gait freezing and frequent falls)	Bilateral *mid-lower* PPN	Significant improvement of Gait and Falls Questionnaire score; no changes in akinesia scores	NA
Thevathasan et al. (2010)	*N* = 11		Significant benefit on gait and balance, but not on akinesia	Moderate improvement in attention (“speed – not accuracy – of reaction” improved with stimulation)

PD, Parkinson’s disease; LFS, low-frequency stimulation; HFS, high frequency stimulation; PPTg, nucleus tegmenti pedunculopontini; PPN, pedunculopontine nucleus; NA, not assessed.

The fascinating proposal that PPN-a-LFS might represent the alternative surgical strategy to manage some Parkinsonian motor symptoms inadequately responsive to traditional DBS targets, has not been confirmed in full (Stefani et al., 2007; Ferraye et al., 2010; Moro et al., 2010b). Recent clinical follow-ups at 2–3 years reported some uncertainties (Moreau et al., 2009; Peppe et al., 2010) or a progressive decline of the transient gait amelioration (in support of likely placebo effect, see Stefani et al., 2009). Despite these inconsistencies, Mazzone and co-authors have documented lately a solid procedure in more than 15 patients mono-laterally implanted in PPTg (thus, a procedure not including the simultaneous targeting of STN – consider Table 2 in Mazzone et al., 2011, from patient 7 to patient 23, plus additional 5 more in recent months); hence, more extended follow-up in a larger patient sample is ahead.

Our first observations (Stefani et al., 2007), focused on the PPTg-LFS (10–25 Hz) in PD patients *simultaneously implanted* in STN, showed some disappointing motor results (at least if compared to 130–185 HFS-STN), but opened new interesting perspectives in terms of beneficial impact on PD non-motor symptoms (NMS), including sleep, attention, and cognitive domains (Alessandro et al., 2010; Costa et al., 2010).

In the last decade, huge efforts in the neurological community have been devoted toward detection and classification of NMS in PD patients, since recognized as a detrimental milestone, which may dictate patient quality of life (Chaudhuri et al., 2006; Hinnell et al., 2012). Accordingly, nowadays the majority of on-going, long-lasting clinical trials include measures of global cognitive functioning and psychopathological profiles, especially if designed to assess the efficacy of complex therapeutic strategies such as DBS (Witt et al., 2008, 2011). Interestingly, in PD patients undergoing STN-DBS, the best motor response was not necessarily correlated to the increase of metabolic activity in frontal cortical areas governing executive functions. STN-DBS was found to partly restore physiologic glucose consumption in limbic and associative projection territories of the basal ganglia, but erratically (Hilker et al., 2004). Overall, a minimal decline – albeit frequently infra-clinical – of cognitive and executive functions may occur after STN-DBS (Parsons et al., 2006; Witt et al., 2008; Daniels et al., 2010). This figure is hard to attribute in full to the STN-HFS *per se*, given the natural PD history, which implies: (a) a continuous decline of dopaminergic function as the disease progresses, independently of pharmacological or surgical treatments (Hilker et al., 2005) and (b) the progressive involvement of other pathways (noradrenergic and cholinergic ones).

This scientific field around PPN-a is largely perturbed by a long-lasting debate concerning appropriateness and functional anatomy of the brain-stem targeting, as we have presented (Mazzone et al., 2005, 2008, 2011; Stefani et al., 2007). In our patients, the stimulation target did in fact correspond to the caudal pontine representation of PPN, which is PPTg (Mazzone et al., 2008; Insola et al., 2012) and, likely, coincides, at least in part, with PPN pars compacta (PPN-C, see Figure 1 in Benarroch, 2013). Contenders have questioned this localization; the Queen’s Square group proposed, for instance, a careful approach to identify an *ideal* stereotactic localization of the nucleus (Zrinzo et al., 2008). At present, the perfect PPN-DBS surgical strategy still waits for further investigations, and we may hope that the academic discussion avoids some crude, occasionally too informal a tone, recently topping the standard dialectics (Aviles-Olmos et al., 2011; Mazzone et al., 2012a).

In the patient’s interest, any group is expected to provide unequivocal correlations between target details and clinical scores. Mazzone et al. (2011) have recently addressed the potential ambiguities via a careful reassessment of the target, based upon an unconventional reconstruction, which overcomes the routine CA-CP landscape and focuses on dedicated landmarks of the brain-stem.

Also the Grenoble group provided a patient-by-patient MRI identification of the target (Ferraye et al., 2010), but acknowledged the variability of the clinical response and the lack of a strict correlation between catheter positioning and gait performance. The French school had initially utilized micro-recording through a rostro-caudal trajectory starting from CA-CP line toward the “para-cuneiform” region, in which a generous “mimicked gait”-firing discharge occurred (consider Piallat et al., 2009). However, similar electrophysiological hallmarks are not shared by others.

For the time being, at least five groups worldwide have chased the PPN-a as potential target (Table 1). Unfortunately, the lack of common protocols has hampered from the beginning the chance to acquire solid conclusions despite the small number’s series. In particular, cognitive evaluations are not always available or appear as anecdotal. Also in terms of the motor outcome, so far, results are inconclusive.

In this scenario, the modest, but consistent, cognitive amelioration induced by PPN-a-LFS, at least in our patients, is of interest, representing the core of the following chapters.

## PPN-Area DBS and Sleep

Pedunculopontine nucleus area is undoubtedly involved in controlling alternations between behavioral states as the result of promoting the thalamo-cortical activation that is thought to regulate both wakefulness and rapid eye movement (REM) sleep in mammals (Rye, 1997; Pahapill and Lozano, 2000). Not surprisingly, different research groups, who have experienced PPN-DBS in an attempt to improve Parkinsonian motor symptoms, have ascertained the impact of 10–70 Hz PPN-stimulation on patients’ sleep quality.

In our hands, PPTg stimulation proved a potent effect on patient sleep architecture, as originally emphasized by a pioneering Polysomnography (PSG) study (Romigi et al., 2008), whose results were subsequently replicated (Alessandro et al., 2010). In these studies, we investigated the specific effect of bilateral PPTg-DBS on sleep structure of two PD patients by means of PSG recordings, which were performed before and after surgery (STN-ON/PPTg-OFF versus PPTg-ON/STN-OFF). The PSG data documented that, before surgery, PD patients featured a severe sleep architecture disruption, with frequent occurrence of nocturnal awakenings, increased Stage I sleep and wakefulness after sleep onset (WASO), plus a remarkable reduction of REM sleep time. In comparison with pre-surgery, both bilateral STN-DBS and PPTg-DBS induced the striking improved of sleep efficiency, with reduction of WASO and nocturnal awakenings, together with a mild reduction of Stage 1 and increase of Stage 2 sleep. However, whereas STN-DBS had no impact on REM sleep, only PPTg-DBS promoted a reduction of REM latency and a relevant increase in REM sleep time (Romigi et al., 2008; Alessandro et al., 2010).

These first PSG findings were soon confirmed by a successive electrophysiological study carried out in three PD patients and two patients affected by progressive supra-nuclear palsy (PSP) undergoing unilateral PPN-a DBS for prominent extra-pyramidal axial signs (Lim et al., 2009). In all those patients, it was found that PPN-a DBS, at both higher (70 Hz in PSP patients) and lower (5–30 Hz in PD patients) frequency stimulation, increased REM sleep time and REM percentage by inducing more REM sleep periods, possibly lowering the REM sleep threshold.

Although the local neuronal effects of DBS are still not fully understood, the physio-pathogenesis of these PSG results may be explained by considering that REM sleep is regulated via reciprocally inhibitory REM “ON” and REM “OFF” nuclei, located into the pontomesencephalic tegmentum (McCarley and Hobson, 1975), and that the PPN-a may promote REM by modulating a brain-stem “flip-flop” switch, represented by the REM “ON” sublaterodorsal and precoeruleus regions, and the REM “OFF” lateral pontine tegmentum and ventrolateral periaqueductal gray (Lu et al., 2006). In these patients, the active stimulating electrodes were located *into* or *near* the PPN *pars dissipata*, and the frequency of stimulation utilized was relatively low (5–70 Hz). Hence, it has been inferred that PPN-a-ON may act on REM sleep by increasing output from the REM “ON” PPN neurons, and trans-synaptically inhibiting the REM “OFF” ventrolateral periaqueductal gray (dorsomedial to PPN) and lateral pontine tegmentum (lateral to PPN) region. In fact, LFS, in contrast with HFS, could increase the firing discharge of neurons next to the electrode (i.e., REM “ON” neurons), and excites, at the same time, inhibitory pre-synaptic afferents to neurons at some distance from the electrode (i.e., REM “OFF” neurons) (Johnson et al., 2008). Moreover, an electrical coupling between PPN-a neurons and both the parafascicular intralaminar nucleus and the subcoeruleus nucleus, equally involved in cortical activation and REM sleep regulation, has been recently demonstrated as a novel mechanism for sleep-awake control. The finding of electrical coupling in a specific area of the reticular activating system endorses the concept that this underlying process, behind specific neurotransmitter interactions, modulates the firing activity across different cell populations to induce changes in sleep-wake state (Garcia-Rill et al., 2007; Heister et al., 2007).

It is well known that PPN-a, part of the reticular activating system, is characterized by powerful ascending cholinergic projections to the associative and non-specific intralaminar thalamic nuclei, counting the centrolateral and the parafascicular neurons (Kobayashi and Nakamura, 2003; Parent and Descarries, 2008), which participate in modulating waking and REM sleep (Datta, 2002; Datta and Siwek, 2002). Within the thalamo-cortical systems, the PPN-cholinergic neurons, characterized by low (less than 10 Hz) and high (20–80 Hz) rates of spontaneous tonic firing, exhibit their maximal firing activity during both REM sleep and waking, which are associated with desynchronization of the electroencephalogram (EEG) (Steriade et al., 1990a,b; Datta et al., 2002). On the basis of PPN-a specific firing properties, intracellular recordings in animal models identified different types of thalamic projecting PPN-cholinergic cells, which are strictly related to the genesis of the ponto-geniculo-occipital (PGO) waves, and thus to the generation of REM sleep and REM sleep dependent cognitive function (Steriade et al., 1990b; Datta et al., 2002; Datta, 2006). Indeed, PGO waves are recorded during and immediately before REM sleep from the pontine reticular formation, lateral geniculate, and occipital cortex and represent the neurophysiological index of mammalian REM sleep (Callaway et al., 1987). Intracellular injection of cholinergic agonists (and/or kainate receptor) demonstrated that kainate receptor agonists were able to increased REM sleep, and at the same time, to generate PGO waves as a consequence of the increased activity of PPN-cholinergic neurons (Datta, 1997; Datta et al., 2002).

Aside these experimental studies, a recent elegant electrophysiological study carried out in a PD patient undergoing to unilateral PPN-a DBS (Lim et al., 2009) supported the evidence that PPN-stimulation exerts a possible REM-promoting effect. In this patient, phasic potentials were simultaneously recorded from the intrapontine depth DBS electrode and the scalp electrodes (24-h video-PSG). These potentials have been identified exclusively before and during the REM sleep, being partially associated to the eye movement. Moreover, their morphology, temporal distribution, and localization were similar to those of PGO waves occurring in other mammals, since their origin was recognized in a restricted region of the pontomesencephalic tegmentum and were invariably followed by characteristic cortical potentials with a latency of 20–140 ms. Taken together, these electrophysiological data support the hypothesis that these waveforms correspond to the pontine component of humans PGO waves and that the PPN-a represent the PGO transferring regions (Datta, 1997).

In a recent clinical study, performed in two PD patients undergoing bilateral PPN-a DBS, it was observed that in awake state PPN-a at 80 Hz induced sleep (Arnulf et al., 2010). Therefore, to substantiate this clinical observation, a daytime PSG was performed at different stimulator settings maintained for at least 5 min each with a 3-min stimulator deactivation between changes in settings. PPN-a-LFS (10–25 Hz) promoted wakefulness in the two patients, while PPN-a-HFS (80 Hz) induced sleepiness and, thus, non-REM sleep (within 0.5–8 min). These data indicate that LFS and HFS of PPN-neurons strongly modulate the activity of the nucleus and its connections, resulting in opposite effects on sleepiness and alertness. Unexpectedly, the same study also showed that in one out of the two patients, the brisk ending of PPN-a-LFS caused fast sleep onset and REM sleep within 3–6 min, which lasted 2.6–9 min, to be followed by spontaneous wakefulness. The precise origin of the unexpected of REM sleep induction after abrupt withdrawal of LFS remains unclear, although the authors stated that a more dorsal placement of the stimulator electrode took place (Arnulf et al., 2010).

In a more recent study, we have investigated further the long-lasting effects of PPTg-DBS (the definition chosen for our target, as suggested in the introduction) on nocturnal sleep and diurnal somnolence in five PD patients (Peppe et al., 2012). Of course, they are still the patients of the original series (neurosurgical procedure including targeting in both PPTg and STN, Stefani et al., 2007).

**Figure 1:**
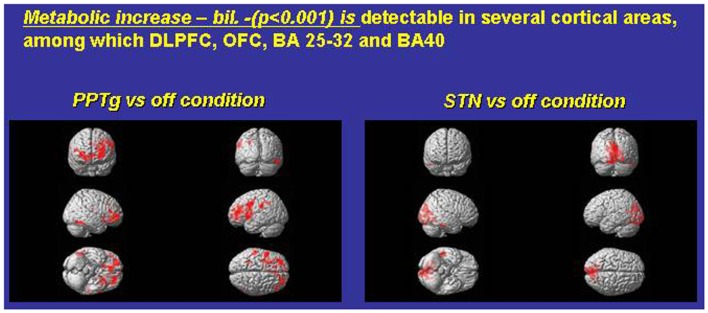
**Exemplary FDG consumption comparing, in the same PD subjects, the condition *only* PPTg-ON (left panel) versus the condition STN-ON (imaging were performed in Pisa, under the supervision of Dr. Ceravolo and Dr. Volterrani; for details, see Ceravolo et al., 2011)**.

**Figure 2:**
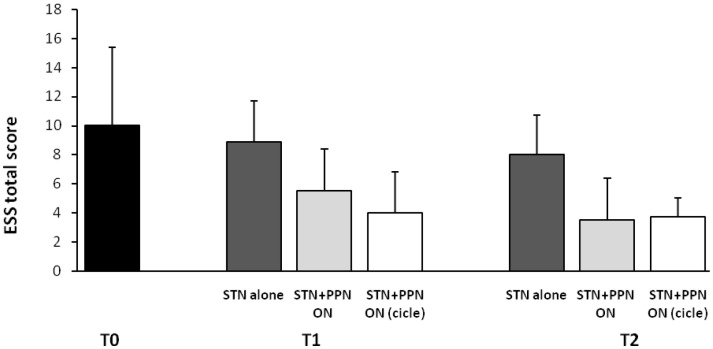
**Shown are mean results of Epworth sleepiness scale (ESS) in original PD patients implanted a long-time ago (2006–2007) in both STN and PPTg**. Histograms refer to before surgery (T0), then 3 (T1), and 12 months (T2) following surgery completion. A modest but significant amelioration of diurnal drowsiness is detected when PPTg-ON is introduced (for details, see Peppe et al., 2012).

This latter analysis was focused on subjective sleep scales, including the Parkinson’s disease sleep scale (PDSS), a specific and comprehensive pragmatic clinical tool designed to address the multi-factorial nature of sleep disturbances in PD (Chaudhuri et al., 2002), and the Epworth sleepiness scale (ESS), an eight-item self-reporting questionnaire assessing symptoms of diurnal drowsiness (Johns, 1991). Following the study design, scales were administered a week before surgery and then 3 months and 1 year after surgery. Maintaining constantly ON the STN-DBS for ethical concerns, the investigation required three different patterns of PPTg-DBS, each kept for 15 days: (1) condition OFF; (2) condition ON (PPTg stimulated 24 h a day); condition CYCLE (PPTg stimulated 12 h at night). The most interesting results can be summarized as it follows: (i) a significant improvement of nocturnal sleep quality was observed at 3-month follow-up and, then, confirmed at 1-year follow-up in comparison to pre-surgery (true in all DBS conditions, although prominent during PPTg cycle stimulation; (ii) only PPTg-DBS, unlike STN-DBS, induced a remarkable long-term improvement of patient daytime sleepiness, as testified by the significant reduction of ESS score at 1-year follow-up (Figure 2); (iii) the cyclic PPTg-DBS proved more efficacious than the continuous one on some aspect of nocturnal sleep, including nocturnal restlessness and psychosis (more details on PDSS and ESS data can be found in Peppe et al., 2012). For the scopes of this review, it is noteworthy that even the Pittsburgh sleep quality index (PSQI), a self-rated clinical scale considered an indicator of relevant sleep disturbances in general population (Buysse et al., 1989), proved the positive influence of PPTg-DBS on sleep-awake cycle. In fact, PSQI showed a remarkable and long-lasting improvement of patient global sleep quality after surgery, mostly noticeable during PPTg-DBS, as demonstrated by the significant reduction of PSQI score observed after surgery (Figure 1). Based on the different PPTg stimulus modalities explored, we indicated the importance of stimulating this area during sleep, showing that cyclic stimulation of the PPTg reveals a better efficacy on PD patients’ sleep quality than the continuous one. Hence, we hypothesized that the amelioration of sleep-awake cycle, clearly evident after the 3-month follow-up (but more under CYCLE), could be due to greater physiological stimulation of PPTg activity. The fact that we found the same results (similar impact on daily somnolence) with both stimulation (ON and CYCLE), at the 1-year follow up, suggests a sort of ceiling effect. Finally, considering that the same two patents who underwent PSGs study, showed a remarkable reduction of EES score during PPTg-DBS, it is interesting to hypothesize a relationship between the improvement in daytime sleepiness and the amelioration of REM sleep, especially considering the PPTg central role in promoting arousal in addition to REM sleep (Rye, 1997).

In conclusion, few studies have been performed in PD patients to assess the objective and subjective changes in sleep-awake cycle related to PPN-a DBS. Moreover, comparison of data is difficult since they were carried out in a small number of subjects, following different surgical approaches and electrode placement and using diverse stimulus parameters. Nevertheless, apart from possible discrepancies in patients’ motor outcome, there is a converging evidence that PPN-DBS exerts a positive effect on sleep quality and wakefulness as well as on nocturnal REM sleep, indicating the central role of PPN-a in sleep physiology and sleep dysfunction in humans, as previously documented from experimental studies.

Noteworthy, and despite our fascination around PPTg-mediated effects on sleep, several therapeutic agents exist already for advanced PD patients suffering alterations of sleep architecture, and DBS should never represent a short-cut indication for this sort of impairments.

## PPN and Cognition

In our hands, PPTg-DBS, originally conceived as a strategy for PD gait impairment, revealed a little influence on postural instability and gait impairment, but regularized sleep and influenced cognitive performance. As a matter of fact, the contribution of PPTg-LFS in changing gait parameters was not significant with respect to STN-HFS (Moreau et al., 2009; Peppe et al., 2010). Yet, both Toronto’s and Grenoble’s groups found more appealing and consistent results in their patient cohorts, with a single mono-lateral target (Ferraye et al., 2010; Moro et al., 2010b).

Our initial observations (Mazzone et al., 2005, 2007; Stefani et al., 2007) concerned PD patients under a double target protocol (PPTg plus STN) and the following biases are possible: first, the implantation of multiple electrode catheters might contribute to severe stereotactic “lesion-effect” (yet, negligible in the long run); second, and more critical, is a sort of tautology: that the enrollment criteria utilized once, in recruiting patients for such a novel and potentially risky surgery, were influenced more by patient’s motivation and absence of co-morbidity rather than the severity of LD-resistant sings (i.e., axial) and or the presence of freezing of gait (FOG, see Stefani et al., 2007). At least two of our first patients were indeed recruited *despite* disease duration <10 years and *despite* scarce FOG. In other words, how could it be possible to judge the specific PPN-mediated influence on FOG in PD patients manifesting only a modest degree of axial akinesia and/or the rare incidence of falls (whilst it was statistically more pronounced in PD confined to the wheelchair or experiencing several weekly falls)? Accordingly, it is still possible, as advocated by others, that the PD patients with an extremely severe postural imbalance and an akinetic profile less responsive to conventional drug therapy may be ideal candidates for surgery aimed at implanting the pontine structures. Moro et al. (2010b) have shown convincingly the clear reduction of falling episode in the 1-year follow of mono-laterally implanted PPN. In the Grenoble series, gait analysis results were also promising (Ferraye et al., 2010, 2011). Mazzone is proceeding with an extensive series of mono-lateral PPTg and gait results seem more consistent (personal communication).

For the purpose of this review, PPTg-LFS, in our patients, proved to have a potent effect on degree of attentiveness and some specific cognitive performances. At odds with some adverse events observed following STN-DBS (i.e., worsening of semantic and phonemic fluency), PD patients under PPTg-LFS may experience a significant amelioration of delayed recall, executive functions and verbal fluency (Alessandro et al., 2010; Costa et al., 2010; Ceravolo et al., 2011).

As recalled (Mazzone et al., 2011), “PPN lesion, in animals, impairs attention, executive function, working memory (WM), and learning” (on this issue, consider the pioneering review by Anderson et al., 2006; Winn, 2006). However, anatomists suggested that an abundant 30% PPN already degenerate in the early PD disease stages; apoptotic-like processes affect the PPN neuropil and may represent a hallmark of extra-pyramidal syndrome. Hence, how is it possible that DBS of a degenerated area is promoting *any* effect? Reasonable answers include the preferential activation of PPN-fugal axonal fibers and the maintained functional roles of surviving PPN neurons (Scarnati et al., 1987; Florio et al., 2007).

The relevance of these findings emerges if compared to the cognitive outcome of standard targets. Careful neuropsychological examination in STN-implanted subjects may in fact disclose a large percentage of subtle deficits in the post-surgery follow-up. Some authors, at one extreme, demonstrated that >30% of STN-DBS patients experienced a cognitive decline sufficient to identify conversion to dementia (Williams et al., 2011). This finding represents a warning, although it did not reach significance with respect to PD not submitted to neurosurgery (Williams et al., 2011).

Are these deficits really and promptly impacting any patient’s quality of life? Prolonged trials, conducted on patients implanted also at younger age (consider the series by Castrioto et al., 2011 and see Vitek, 2012) or preliminary data from the *early stim* group, a multi-center investigation on about 150 early onset PD, suggest not overestimating this issue (Schuepbach et al., 2013).

Nevertheless, an honest reappraisal of the literature about STN-DBS, even if performed by unbiased experts, admits the potential increase of apathy, and some degree of impairment in non-verbal recall, oral information processing speed, and semantic fluency (Bronstein et al., 2011).

In this content, the PPN-mediated cognitive benefits are remarkable, despite the small cohort and the relatively modest effect.

We found no significant changes between pre-surgery and PPTg-OFF/STN-OFF conditions (Ceravolo et al., 2011). Conversely, the condition PPTg-ON/STN-OFF significantly improved cognition in different domains when compared to PPTg-OFF/STN-OFF. PPTg-ON ameliorated verbal long-term memory, assessed with the California Verbal Learning test delayed recall (*p* < 0.025), and executive functions, as revealed by both TMT (B-A *p* < 0.025) and verbal fluency (*p* < 0.001). Therefore, we did conclude PPTg-DBS “fueled a significant cognitive improvement” (Ceravolo et al., 2011).

Costa et al. (2010), in another study (Table 1), investigated the WM functioning in the same subjects (five PD patients who underwent simultaneous PPTg- and STN-DBS). PD patients were evaluated (in the morning at least 12 h after antiparkinsonian therapy withdrawal) comparing two conditions: (i) after continuous PPTg stimulation (OFF Therapy/ON PPN); (ii) 120 min after PPTg had been switched “OFF” (OFF Ther/OFF PPN). The experimental WM task consisted of an n-back paradigm with verbal and visual-object stimuli. PD patients manifested “a consistent response time decrease on both the verbal and the visual-object tasks under the PPTg-ON condition (*p* < 0.05).” However, the accuracy score did not significantly differ between the two experimental conditions. It was suggested that PPTg-stimulation “facilitates the speed of processing of information in the content of WM, possibly through the modulation of the attentional resources” (Costa et al., 2010).

As a corollary study, Zanini explored the possibility that combined STN-HFS and PPTg-LFS influenced language. This study observed “an interesting trend toward reduction of ungrammatical errors (particularly substitution of free and inflectional morphemes)” when stimulating either STN (at 130 Hz) or PPTg at 20 Hz (Zanini et al., 2009; Table 1).

Which mechanisms may underlie this surprising pattern of effects? Figure 1 compares paradigmatic PET, performed on the same PD subject, before and during either STN-ON (180 Hz) or PPTg-ON (25 Hz) (Figure 1). Such an extended and bilateral activation of several cortical areas (including medial frontal structures) is intuitively detected. The most relevant findings could be summarized as follows:

- An extensive and bilateral metabolic activation of several cortical regions, including both medial and dorso-lateral area of the frontal lobe (Figure 1);
- The mono-lateral activation of the ventral striatum (Figure 1);
- The decrease of metabolic activity in the left anterior cerebellum (culmen, not shown) (Alessandro et al., 2010; Ceravolo et al., 2011).

However, to date, other groups, utilizing since the late 2000s a mono-lateral (and more medial) target inside PPN-a, emphasized rather different metabolic results (acquired with different technique). Strafella and co-authors described mostly a PPN-mediated increase of metabolic activity – *rCBF changes* – on viciniori GPi and thalamus (Strafella et al., 2008; Ballanger et al., 2009).

A possible limitation of our studies concerns the lack of comparative evaluations of the condition PPTg-ON versus pharmacological ON-state. Another bias could derive from the fact that few consistent data are available, at present, on low-frequency STN-related changes of cortical metabolic activity. Hence, there is not sufficient ground to exclude, albeit unlikely, that a low-frequency STIM of STN might re-propose metabolic changes similar to PPTg (Stefani et al., 2010). A very recent contribution suggested, in four patients with bilateral PPN and caudal zona incerta (cZi) DBS electrodes that combined PPN/cZi stimulation produced a statistically significant improvement in UPDRS-III score compared to cZi stimulation alone, but only in the condition MED-ON (Khan et al., 2012). Interestingly, “the concomitant PPN/cZi stimulation had a cumulative effect on levels of rCBF, effectively combining sub-cortical and cortical changes induced by stimulation of either target in isolation” (Khan et al., 2012).

## Conclusion

Limited experience worldwide impedes the drawing of conclusive statements on the clinical efficacy of PPTg stimulation on both motor and non-motor domains. Any straightforward assumption is, in our opinion, quite presumptuous. Major effort should be launched for assessing to what extent the impact on sleep and cognition, as detailed in our hands, may in fact be replicated by other teams utilizing different trajectories; for the time being, whether some of the NM benefits described (i.e., changes of sleep structure) are really target-dependent and/or, at least in part, frequency-dependent, might be addressed. In addition, the significance of clinical changes in cognitive performance need to be evaluated in light of real changes in routine functional way of life (as explored on dementia in Stefani et al., 2013). On-going studies and extended follow-up will contribute at clarifying this issue.

Unfortunately, as we acknowledged, the current technologies do not support the attribution of a specific effect to any specific contact lead and it is difficult to speculate on which sub-cortical pathways (Aravamuthan et al., 2007, 2008) or spinal circuitry is involved, since the commercially available catheters affect a-specifically a plethora of *en passant* circuits. Yet, and *despite* rather different targets, the LFS of the “PPN region” implanted by Bristol (Khan et al., 2012) and Rome (Ceravolo et al., 2011) seems to provide quite similar impacts on cortical metabolism.

With the present procedures, nobody has ascertained, so far, to what extent stimulation of PPN preferentially modulates and/or activates specific neuronal phenotypes (gabaergic, glutamatergic, cholinergic) or fibers.

That said, the pattern of responses detected in our original cohort may be attributed to more selective activation of the pontine cholinergic contingent (abundant in PPN-C/PPtg) and explain the robust effect on sleep and behavioral state. In contrast, a more selective activation of dorsal and medial sub-regions (likely targeted by Toronto’s and Grenoble’s series) may indeed reflect into discrete regional activation of nearest basal ganglia stations (Strafella et al., 2008) and solid effects on falls prevention (Ferraye et al., 2010; Moro et al., 2010b; Thevathasan et al., 2012).

One critical aspect to mention is that the PPN region, in PD patients as well as in atypical Parkinsonism, is affected by severe neuro-pathological abnormalities; a loss of a 30–40% neurons is supported by post-mortem studies. We are tempted to speculate that large differences in clinical response might be attributed not to neuro-functional targets *per se* but also to neuro-pathological differences between patients, for example in terms of the severity of cholinergic neuronal loss, correlated in turn with the severity of axial symptoms (Rinne et al., 2008).

In other words, some discrepancies between groups (or even in the same patient’s series) are possibly explained by the different degree of PPN neuronal loss as manifested along the ponto-mesencephalic axis in each patient submitted to surgery. We know that the “the activity of thalamic-projecting cholinergic neurons of the PPN is consistent with their role in inducing and maintaining thalamo-cortical activation associated with desynchronization of the EEG during wakefulness and REM sleep” (Steriade et al., 1990; Benarroch, 2013). What we do not know, of course, is the abundance of the residual not degenerated contingent of fibers potentially activated by LFS in each of our patients.

Although results between groups appear divergent in many respects, 6 years experience on PPN-a/PPTg stimulation, in humans, corroborates the following statements:

1. Clinical results support the hypothesis that single, mono-lateral PPTg-LFS is advisable and safe, as originally advocated by Toronto’s group (Moro et al., 2010b);
2. The “pro-attentive pattern,” promoted by PPTg-ON in our first series, is very peculiar if compared to detrimental or negligible cognitive effects promoted by routine STN-HFS. This may suggest that endogenous transmitters other than catecholamines are involved when PPTg (and PPN-a in a broad sense) was stimulated. In our opinion, a widespread activation of ascending pathways (mostly cholinergic fibers toward intra-thalaminar nuclei) is the key factor. The unequivocal demonstration would demand verification, through micro-dialysis, of the on-going release of Ach or catecholamines or the modulation of trophic factors and/or nucleotides in main target areas of PPN-a (such as GPi/SNr on one hand and CM/Pf on the other) (see for example Fedele et al., 2001; Stefani et al., 2005, 2011).
3. The relevance of the PPTg-mediated benefits on the NM spectrum signs is intriguing and supports, albeit speculatively, the working hypothesis that the stimulation of specific sub-portions of the pontine tegmentum should be tested as a therapeutic chance in severe extra-pyramidal syndrome, such multi-system atrophy (Brusa et al., 2009; Mazzone et al., 2012b), or neurological states dominated by vigilance defects.

Our original contention – PPTg as a potential trigger to fuel endogenous circuitries targeting large thalamic and cortical areas – might be either validated or decline in a few years, provided that a combination of technically refined approaches, adjourned peri-surgical imaging, better application of neuro-physiological hallmarks in the postsurgical phase and data acquisition from larger series take place.

## Preliminary Disclosure

This manuscript does not represent an evidence-based review and, of course, it contains many subjective opinions. Only a larger observations, a more extended follow-up and, possibly, shared neurosurgical parameters, would allow, in the next future, more clear conclusions.

## Conflict of Interest Statement

The authors declare that the research was conducted in the absence of any commercial or financial relationships that could be construed as a potential conflict of interest.
